# Vitamin D and Calcium Insufficiency-Related Chronic Diseases: an Emerging World-Wide Public Health Problem

**DOI:** 10.3390/ijerph6102585

**Published:** 2009-10-02

**Authors:** Meinrad Peterlik, Steven Boonen, Heide S. Cross, Christel Lamberg-Allardt

**Affiliations:** 1 Department of Pathophysiology, Medical University of Vienna, Waehringer Guertel 18–20, A-1090, Vienna, Austria; E-Mail: heide.cross@meduniwien.ac.at; 2 Center for Metabolic Bone Diseases and Division of Geriatric Medicine, Leuven University, Universitaire Ziekenhuizen, Herestraat 49, B-3000, Leuven, Belgium; E-Mail: steven.boonen@uz.kuleuven.ac.be; 3 Department of Applied Chemistry and Microbiology, Calcium Research Unit, University of Helsinki, Agnes Sjobergin katu 2, F-00014 Helsinki, Finland; E-Mail: christel.lamberg-allardt@helsinki.fi

**Keywords:** vitamin D status, calcium intake, 25-hydroxyvitamin D, 1,25-dihydroxyvitamin D, calcium-sensing receptor, osteoporosis, colorectal cancer, breast cancer, prevention, food fortification

## Abstract

Vitamin D and calcium insufficiencies are risk factors for multiple chronic diseases. Data from 46 recent studies from Europe, North America, South-East Asia and the South Pacific area clearly indicate that a low vitamin D status and inadequate calcium nutrition are highly prevalent in the general population (30–80%), affecting both genders. The extent of insufficiencies is particularly high in older populations, and in some geographical areas, also in children and in young women of child-bearing age, in ethnic minorities and immigrants, as well as in people of low socio-economic status. Enrichment of cereal grain products with vitamin D and calcium would be a viable approach to increase consumption and improve health outcomes in the general population worldwide.

## 1. Introduction

It is common knowledge that an inadequate supply of vitamin D and calcium has negative effects on bone health at all ages, inasmuch as it causes rickets in infants, retards acquisition of an adequate bone mass during skeletal development in adolescents, and is finally responsible for accelerated bone loss in adulthood in both women and men, leading to the development of osteoporosis. Importantly, there is also evidence from epidemiological studies, clinical intervention trials as well as from studies with animal models of human diseases that a compromised vitamin D status and inadequate calcium nutrition are predisposing conditions for a great number of other diseases, including various types of cancer, chronic infectious, inflammatory and autoimmune diseases, metabolic disorders, as well as hypertension and cardiovascular diseases (Table 1; for details, [1–3]).

## 2. Why a Low Vitamin D Status and a Nutritional Calcium Deficit are Risk Factors for many Chronic Diseases

Vitamin D comes from two sources in humans, it could either be synthesized in form of vitamin D_3_ (cholecalciferol) under the influence of solar UV-B radiation in the epidermis, or be absorbed from the diet or from supplements and food additives, which in some countries may contain vitamin D_2_ (ergocalciferol). In any form, vitamin D is transferred to the liver, where it is metabolized to 25-hydroxyvitamin D (25-(OH)D). The term 25-(OH)D is used to denote the sum of 25-(OH)D_3_ and 25-(OH)D_2_. Thus, the plasma level of this metabolite reflects the sum of vitamin D from endogenous synthesis and from dietary intake, and is therefore a reliable indicator of an individual’s vitamin D status.

**Figure 1.**
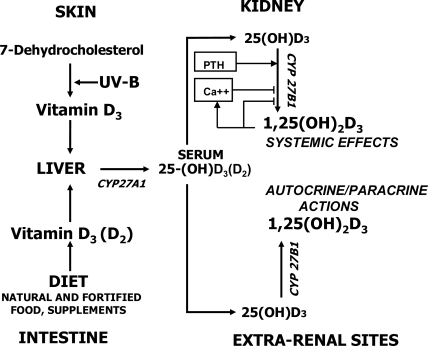
Synthesis, absorption and metabolism of vitamin D.

Conversion of 25-(OH)D_3_ to the biologically most active metabolite, 1,25-dihydroxyvitamin D_3_ (1,25-(OH)_2_D_3_), is catalyzed by the *CYP27B1*-encoded enzyme, 25-(OH)D-1α-hydroxylase, and takes place predominantly in the kidney, but also at many extra-renal sites [4] (see Figure 1): Examples are normal and neoplastic epithelial cells of the skin [5], of the gastrointestinal tract [6,7] and of female and male reproductive organs [8–10] as well as osteoblasts and osteoclasts [11,12] and cells of the vascular [13], the central nervous [14] and the immune system [15,16].

1,25-(OH)_2_D_3_ bound to the nuclear high-affinity vitamin D receptor (VDR) functions as a transactivating regulator of gene expression. Circulating 1,25-(OH)_2_D_3_, which is produced by up to 90% in the kidney, plays a key role in *systemic* calcium and phosphate homeostasis by regulating ion fluxes in its classical target organs, i.e., small intestine, kidney and bone. 1,25-(OH)_2_D_3_ is also an important *local regulator* of cellular proliferation, differentiation and function in many organs and cell systems that express the 25-(OH)D-1α-hydroxylase [4] (Figure 1). The extent of intracellular synthesis of 1,25-(OH)_2_D_3_ at extra-renal sites depends largely on ambient 25-(OH)D_3_ levels and is not associated with circulating 1,25-(OH)_2_D_3_ concentrations (e.g., [17]). Therefore, at low serum levels of 25-(OH)D, 25-(OH)D-1α-hydroxylase activity may not be sufficient to maintain tissue concentrations of 1,25-(OH)_2_D_3_ necessary for efficient autocrine/paracrine regulation of cellular growth and function. This explains why many chronic diseases, as listed in Table 1, show significant negative associations with serum 25-(OH)D [1,3,18]. Importantly, low serum 25-(OH)D has been shown to be a reliable predictor of all-cause mortality [19].

**Figure 2.**
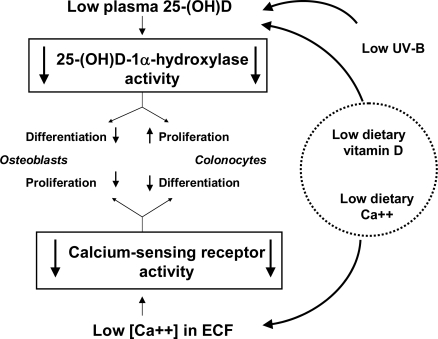
Tissue-specific consequences of low vitamin D and calcium status on cellular proliferation and differentiation. ECF, extracellular fluid.

There is evidence from many human and animal studies for a significant inverse relationship between dietary calcium and risk of multiple chronic diseases [1]. This is difficult to understand because the effect of even large variations in calcium intake levels on extracellular calcium concentrations [Ca^2+^]o is attenuated by the systemic actions of calcium-regulating hormones allowing physiological variations in [Ca^2+^]o to occur only within a narrow range. However, many types of cells express a calcium-sensing receptor (CaR), which senses even minute changes in [Ca^2+^]o and thus allows Ca^2+^ to function as a “first messenger” for various cellular responses [20]. An important feature of the CaR is the high cooperativity between multiple Ca^2+^-binding sites in its extracellular domain. This results in amplification of signals from extracellular Ca^2+^, which, by cell-specific coupling to stimulatory and inhibitory G proteins, are transduced into various intracellular signalling pathways. Expression of a functioning CaR thus allows cell-specific reactions to physiological changes in [Ca^2+^]o. The CaR not only controls PTH secretion from parathyroid gland cells but plays key roles in normal cartilage and bone formation [21–23], as well as in limitation of cellular growth of normal and neoplastic cells [2,24]. Conversely, low dietary calcium causes hyperparathyroidism by impairment of CaR activity and, by the same token, can be linked to the development of not only osteoporosis and various malignancies, but possibly other calcium-insufficiency-related chronic diseases (Table 1) (for details, [1,3]; see also Figure 2).

Relevant for our understanding how vitamin D and calcium status interact in the pathogenesis of chronic diseases, is the observation that most cell types jointly express the 25-(OH)D-1α-hydroxylase and the CaR. Therefore, cell-specific cooperative signalling from 1,25-(OH)_2_D_3_/VDR and CaR, which is necessary to maintain normal cell functions (as detailed in [1,3]), is impaired under conditions of vitamin D and calcium insufficiency (Figure 2). This has been shown particularly for osteoporosis and many malignancies, particularly colorectal and breast cancer (as detailed in the following). Low serum 25-(OH)D and inadequate calcium intake reportedly are associated with cardiovascular risk factors such as hypertension [25–27], obesity [28–30], metabolic syndrome and diabetes mellitus type II [31,32]. Vitamin D and calcium insufficiencies have also been correlated with incident cardiovascular symptoms, including angina, coronary insufficiency, myocardial infarction, transient ischemic attack, and stroke [33], as well as with greater mortality from chronic cardiovascular disease [19,34,35].

## 3. Vitamin D Insufficiency: A World-Wide Phenomenon

### 3.1. Definition of Vitamin D Insufficiency

The vitamin D status of an individual is a composite of UV-B mediated synthesis of vitamin D_3_ in the epidermis and of intake and absorption from the gut. Outright vitamin D deficiency is indicated by plasma 25-(OH)D levels below 10–15 nM [36]. In this situation, 1,25-(OH)_2_D production in the kidney is severely limited because of substrate depletion, causing a decrease in intestinal calcium absorption with rickets or osteomalacia as a consequence. At 25-(OH)D serum concentrations above 15 nM, the kidney produces enough 1,25-(OH)_2_D_3_ to maintain systemic mineral ion homeostasis [37], but availability of 25-(OH)D for intracellular production of 1,25-(OH)_2_D_3_ at extra-renal sites may be insufficient for autocrine/paracrine control of cellular functions [1]. The definition of vitamin D insufficiency is still a matter of debate. At one time, a serum 25-(OH)D concentration of ~30 nM was thought to be the delimitation between vitamin D insufficiency and adequate vitamin D supply [38]. Now there is growing agreement that serum 25(OH)D should be at least 50 nM [39] (see also Tables 2–4). Even higher cut-offs, e.g. 60–100 nM, are supported by studies on optimal health outcomes [40].

### 3.2. Epidemiology of Vitamin D Insufficiency

Considering reasons for vitamin D insufficiency, one has to take into account that cutaneous UV-B-mediated production of vitamin D_3_ is affected by many factors, such as time of the day, season of the year, latitude, altitude, skin pigmentation or use of sunscreens. Also aging can markedly reduce the capacity of the skin to produce vitamin D_3_ [41]. Geographical differences in vitamin D status result from varying contributions to the vitamin D supply from exposure to solar UV-B but also from intake of dietary and supplemental vitamin D [42,43]. Table 2 lists the results of the available nationally representative studies on prevalence of vitamin D insufficiency in the normal adult population in Europe, North America, East Asia and in the South Pacific area.

In Europe, 7–27% of the adult population have a serum 25-(OH)D concentration below 25–30 nM [26,38,44–47]. In South-East Asia and in Australia, incidence of vitamin D insufficiency varies between 8–17% [48–50]. A relatively low value of 5% has been reported for the USA [51]. However, if 50 nM 25-(OH)D is considered the upper reference limit, on the average one-half of the adult population in Europe [26,38,44–47], Western Canada [52], Australia [53,54] and New Zealand [55] presents with vitamin D insufficiency, whereas only one-third is afflicted in the USA [51] (Table 2).

It must be noted that the proportion of the general population with serum 25-(OH)D below the desirable level of 70–80 nM [40,56] is 73% in the USA [51], 84–87% in Europe and in the South Pacific area [44,46,55] and up to 97% in Canada [52].

### 3.3. Vitamin D Insufficiency in Different Population Segments

*Elderly people*: It has been known for decades that vitamin D insufficiency is common in people, who are immobilized because of chronic diseases or are housebound due to old age. However, recent nationally representative data show that vitamin D insufficiency is present in a substantial portion of old age ambulant people worldwide (Table 3): For example, the European SENECA Study on diet and health of elderly people from 19 towns in 12 European countries revealed that overall 36% of men and 47% of women had serum 25-(OH)D concentrations below 30 nM [57]. In the Netherlands, 50% of people aged 65 years and older had serum levels of 25-(OH)D below 50 nM [58]. Similar values were reported for elderly women in Belgium [59]. According to the OPTIFORD Study [60], 50–92 % of elderly women in Denmark, Finland, Ireland and Poland had wintertime 25-(OH)D concentrations lower than 50 nM. On the average, prevalence of vitamin D insufficiency in elderly people in North America [51,61], Australia [62], New Zealand [55] and Japan [63] seems to be lower than in Europe (Table 3).

*Children, adolescents and young adults:* In some European countries such as Denmark, Finland, Ireland and Poland [60], 37% of 12-year old girls had 25-(OH)D concentrations lower than 25 nM (Table 4). By using a broader definition, i.e., <50 nM, 92% had to be considered vitamin Dinsufficient. Comparative values are much lower in Germany [64], but considerably higher in France [65].

In the USA, the proportion of male and female adolescents between 12–19 years, who had 25-(OH)D values below 50 nM, ranged from 24 to 31%, respectively [51]. Incidence of vitamin D insufficiency in younger women is low in Canada [66], compared to Indonesia, Malaysia [67] or Japan [50]. Alarmingly high rates were found in older girls and young women at child-bearing age in India [68] and China [69,70] (Table 4).

*Pregnant women and neonates*: In Europe and in the USA, a poor vitamin D status is observed with increasing frequency in pregnant women [71] and consequently in their neonates causing a high risk of not only rickets, but also non-skeletal diseases in later life, e.g. type 1 diabetes [72].

*Obesity* deserves a special note as a condition frequently associated with vitamin D and calcium insufficiency. A number of earlier studies have well documented a high prevalence of vitamin D insufficiency in morbidly obese women. Now evidence is emerging that in otherwise healthy women and men, body fat mass is frequently inversely associated with vitamin D insufficiency [29,73]. Sequestration of vitamin D in the subcutaneous fat, which alters its release into the circulation, could be one by which obesity could contribute to vitamin D insufficiency [74].

*Ethnic groups:* The immigrant population is at high risk for vitamin D insufficiency in many European countries such as Denmark, Norway and Great Britain [75–77]. In Germany, the proportion of vitamin D inadequacy in children and adolescents, aged 2–17 years, is higher in immigrant than in non-immigrant girls and boys at any time. Notably, after termination of vitamin D supplementation for prophylaxis of rickets between the age of 1–2 years, serum 25-(OH)-D levels fell rapidly below 50 nM in both groups [64]. In the USA, prevalence of vitamin D insufficiency in Mexican American and Non-Hispanic black people is higher than in Non-Hispanic White individuals [51]. Analogous skin pigmentation and degree of vitamin D inadequacy has been reported for three ethnic groups in New Zealand, i.e., Pacific people, Maori and people of European origin [55]. In Australia, dark-skinned and “veiled” women, particularly when pregnant, belong to the group with the highest risk for vitamin D insufficiency [78].

## 4. Inadequate Calcium Intake: A World-Wide Problem

### 4.1. Recommended Calcium Intake Levels

Different intake levels for calcium are recommended by FAO/WHO experts for infants, children and adults [79] to assure optimal whole body calcium retention and consequently adequate development and maintenance of bone mass and mineral density. For children and adolescents between 10–18 years of age, consumption of 1,300 mg per day is recommended, while 1,000 mg per day apply for men between 25–50 years of age and also for women in the same age group, except when higher intake is necessary during pregnancy or after menopause. Recommended calcium allowance per day for males over 65 years and postmenopausal women is 1,300 mg [79].

### 4.2. Epidemiology of Calcium Intake

Findings listed in Table 5 indicate that in Europe daily calcium intake from nutrient sources is consistently low. For example, 84% of the adult population in Austria fail to meet recommended intake levels [44]. The situation is apparently better in Germany [80,81], with one study reporting daily calcium intake even at recommended levels [26]. This seems to be also the case in Great Britain [82].

In contrast, 40% of the population does not meet adequacy in the USA [83]. Similar values probably pertain for Australia [84] and New Zealand [85]. Special consideration must be given to the nutritional calcium deficit in South-East Asian countries such as Indonesia, Malaysia [67] and Bangladesh [86]: The situation is particularly alarming in Bangladesh, where 47% of premenopausal women in the higher socio-economic brackets failed to meet a daily allowance of 400–500 mg calcium, and 63% of women of low socio-economic standing had calcium intake even lower than 200 mg/day [86].

### 4.3. Population Segments with Low Habitual Calcium Intake

*Chronically ill people:* A chronically negative calcium balance due to malabsorption develops, for example, in the many individuals suffering worldwide from lactose intolerance or from inflammatory bowel disease (Crohn’s disease, ulcerative colitis). In addition, calcium malabsorption must be reckoned with in all cases of vitamin D insufficiency resulting from intestinal, hepatic, renal or endocrine disorders as well as in the group of bariatric surgery patients who increase in numbers as a result of the obesity epidemic in the affluent parts of the world.

*Individuals with reduced physical activity:* It must be noted that immobilization even for a short period, e.g. 1–2 weeks of bed rest leads to mobilization of calcium from bone and consequently to net calcium loss [87]. Therefore not only patients in geriatric, psychiatric or neurological care, but also healthy individuals with low habitual physical activity have an increased risk of calcium insufficiency.

*Elderly people:* The data collated in Table 6 confirm the long-standing assumption that particularly the elderly ingest significantly less calcium in their diet than the recommended amount, which is currently considered 1,300 mg per day for this age group [79]. In the European SENECA Study [88], the overall mean calcium intake by elderly people was 894 mg per day, with variations from 600–1,100 mg between different study sites. In the OPTIFORD Study, the median calcium intake among elderly women was 632 mg per day, being lowest in Poland (325 mg) and highest in Finland (925 mg) [60]. In the USA, the mean intake of calcium in women after age 55 is only ~600 mg/day [61]. Daily consumption of ~500 mg by elderly Japanese women [89] is far below a recommended level of 1,200 mg, although daily calcium requirements of East Asian populations may be lower for ethnic reasons [79].

*Children, adolescents and young adults*: In the European OPTIFORD study the median daily calcium intake of girls at a mean age of ~13 years was 823 mg, ranging from 524 mg in Poland to 1,092 mg in Finland [60] (Table 7). Data from the USA indicate that after the age of 10, calcium malnutrition is a common phenomenon. For example, average daily calcium intake in a group of young adolescents (12.7 ± 1.0 yr of age) was found to be 906 mg [90]. Grossly inadequate calcium intake was observed also in young adults in Canada [91]. Average daily calcium intake by schoolgirls in India between 400–500 mg [68], though corresponding to recommended daily allowances for Indians, nevertheless is far below current FAO/WHO recommendations of 1,000–1,300 mg/day [79].

*Ethnic groups:* It has to be borne in mind that not only vitamin D deficiency but also a nutritional calcium deficit is an important cause of rickets [92]. So-called calcium deficiency rickets are prevalent in Middle Eastern and many sub-tropical and tropical countries, such as Nigeria, Ethiopia, South Africa, India and Bangladesh, despite the fact that such countries have ample sunlight [93–95]. Under this condition, the disease is attributable to low dietary calcium intake from mainly cereal-based diets.

## 5. Strategies for disease prevention

Studies on the vitamin D intake in different parts of the world cannot be directly compared because results may be confounded to some extent by differences in life style and clothing habits, consumption of traditional foods or supplement intake. Exact determination of the extent of calcium malnutrition is also difficult, because different methods are used for evaluation of daily calcium intake from nutrient sources and, in addition, for ethnic, dietary and geographical reasons different recommendations apply for different parts of the world [79]. However, combined evidence from all the studies that are included in the present survey clearly indicates that vitamin D insufficiency and calcium malnutrition are common in both genders worldwide, not only in elderly people as previously believed but also in younger adults. Importantly, the highest rates of insufficiencies are found in children and adolescents as well as in women of child bearing age.

### 5.1. Need to Increase Combined Intakes to Daily 800 IU Vitamin D and 1,200 mg Calcium

*Vitamin D:* A recent survey on world-wide vitamin D intake [96] clearly indicates that in many countries vitamin D supply from nutrient sources is too low to sustain mean 25-(OH)D levels in the general population between 40–100 nM, which are considered sufficiently high to achieve a better health outcome [97]. Cashman *et al.* [98] calculated that in 20–40 yr old adults, depending on the extent of sun exposure in summer, daily intakes of vitamin D between 300 and 1,600 IU are required to maintain an adequate vitamin D status in wintertime. Notably, Nelson *et al.* [56] reported that daily doses of 800 IU vitamin D_3_ were sufficient to sustain “optimal” 25-(OH)D serum concentrations (≥75nM) in 80% of a group of pre-menopausal women. With approximately the same daily dose of vitamin D_3,_ serum 25-(OH)D levels could be maintained at 50 nM in 97.5% of a group of elderly people in the absence of sufficient sun exposure [99]. The beneficial effects of 800 IU supplemental vitamin D for various health outcomes are well documented. Daily intake of 700–800 IU vitamin D_3_ maintains normal bone turnover in healthy men at wintertime [100], and reduces the risk for colorectal or breast cancer by 50% [101,102].

*Calcium:* The suggestion to raise daily consumption of calcium to an average of 1,200 mg per day is based not only on physiological considerations [103] but can be deduced also from considerations of optimal health outcomes: For example, daily doses of 1,200 mg calcium effectively prevent osteoporotic bone loss and fractures in people aged 50 years or older [104], and cause a 40–50% risk reduction of colorectal cancer in men and of breast cancer in premenopausal women [105,106].

### 5.2. Rationale for Advocating Combined Intake of Vitamin D and Calcium

Simultaneous correction of nutritional vitamin D and calcium deficits for prevention or amelioration of many chronic diseases is necessary for two reasons: First, dietary intakes of vitamin D and calcium are strongly associated [107] and therefore vitamin D insufficiency is frequently associated with low calcium intake [1,44]. Second, because vitamin D and calcium interact positively in modulation of cellular proliferation, differentiation and function, as detailed before [3,18], it can be expected that an adequate vitamin D status is required to achieve the nutritional benefits of calcium and *vice versa.*

*Osteoporosis:* Combined supplementation with daily 800 IU vitamin D and 1,200 mg calcium is the essential basis for pharmacological prevention and treatment of osteoporosis. From a meta-analysis of 10 randomized controlled trials of oral vitamin D with or without calcium supplementation *vs* placebo/no treatment on the risk of hip fracture in elderly people, Boonen *et al.* [108] concluded that oral vitamin D appears to reduce the risk of hip fractures only when calcium supplementation is added.

*Cancer*: Lappe *et al.* [109] reported evidence from a randomized placebo-controlled trial that in post-menopausal women combined high-dose calcium and vitamin D_3_ supplementation, i.e., 1,400–1,500 mg calcium *plus* 1,100 IU vitamin D_3_, reduced the cumulative risk of cancer of the breast, lung, colon, uterus, lymphoid and myeloid system to 0.232 after four years of trial. Cho *et al.* [110] concluded from an analysis of pooled primary data from 10 cohort studies with a follow-up of more than half a million individuals for 6–16 years, that optimal risk reduction for colorectal cancer necessitates high intake levels of both vitamin D and calcium. This notion was shown to be valid not only for Western but also for Asian populations [111]. In pre-menopausal women, Bérubé *et al.* [112] found highly significant inverse relations between total intakes of vitamin D and calcium and breast density, which is a surrogate marker for breast cancer risk. It is noteworthy, that higher intake of one nutrient was related to lower breast density only in the presence of higher intake of the other nutrient.

## 6. What can be Done?

Simultaneous supplementation of vitamin D and calcium represents a safe and inexpensive strategy for prevention of osteoporosis, colorectal and breast cancer and possibly of many other chronic diseases (Table 1). It must be emphasized that daily consumption of 800 IU vitamin D and 1,200 mg calcium is well below the currently accepted tolerable upper intake levels of 2,000 IU (=50 μg) vitamin D_3_ and 3,000 mg calcium [79].

### 6.1. Supplementation by Fixed Vitamin D/Calcium Combination Tablets

Osteoporotic fractures can be effectively prevented at relatively low cost by combined supplementation with 1,200 mg/d calcium and 800 IU/d vitamin D_3_. However, a significant effect of vitamin D/calcium treatment is only seen in cohorts with at least 80% compliance [104]. Low compliance and lack of adherence seen with any long-term medication certainly will limit the usefulness of combined vitamin D and calcium supplementation for correction of respective insufficiencies in the general population. Therefore, combined vitamin D and calcium supplementation should be promoted specifically for disease prevention in high risk groups, i.e., individuals, who otherwise are unable to attain a normal vitamin D and calcium status due to specific living conditions (immobilization, physical incapacitation, advanced age, chronic diseases), traditional or personal nutritional habits, preferred lifestyle (lack of physical activity, indoor dwelling) etc.

### 6.2. Vitamin D and Calcium Enrichment in Single Foodstuffs

Fortified foods are an important source of vitamin D for those who consume them [113]. In a recent survey on the efficacy of food fortification on serum 25-(OH)D concentrations [114], dose-effect relations for vitamin D from food sources were found equivalent to those reported for vitamin D supplements. Vitamin D-fortified milk has been found to be a safe, effective and acceptable method of administering vitamin D, particularly to the elderly, community-based population. Orange juice fortified with vitamin D_2_ (1,000 IU/240 ml) was tested for its suitability to serve as an alternative for vitamin D-fortified milk. Fortification with vitamin D_3_ of wheat and rye bread is technically easy, and stability and bioavailability of vitamin D is good [115]. Consumption of bread fortified with 5,000 IU vitamin D_3_ and 320 mg calcium per daily serving for 12 months improved the vitamin D status of sun-deprived nursing home residents. Together with suppression of secondary hyperparathyroidism this apparently caused a significant increase in bone mineral density at the lumbar spine and the hip [116].

Calcium fortification is in use all over the world: Staples and food stuffs that are enriched with calcium include flour, cereals, milk, orange juice, mineral waters, soymilk etc. [117]. Calcium- and vitamin D-fortified milk, when providing 800 IU vitamin D and 1,000 mg calcium per day, has a significant positive effect on bone mass and strength in older men [118,119].

It is apparent that fortification of traditional and widely consumed foodstuffs (milk and milk products, bread, orange juice etc.) will guarantee a minimum additional supply of vitamin D and calcium.

### 6.3. Vitamin D and Calcium Addition to Cereal Grain Products

At present, cereal grain products, such as flour, corn meal, noodles and the like, are enriched with vitamin D and calcium in some countries in Europe and in the USA. Newmark *et al.* [117] summarized the rationale, data, efficacy, safety, cost and practicality of the addition of both calcium and vitamin D to cereal grain products to reduce the risk of osteoporosis and colon cancer. The authors estimate that, if cereal grain products were uniformly enriched with 90 IU/100g vitamin D, average daily intake of vitamin D could increase by up to 200 IU vitamin D. Enrichment of cereal grain products with 1,200–1,800 mg/kg calcium could raise dietary intake by about 200–400 mg/day. A conservative estimate suggests that through these measures at least a 20% reduction of the rate of osteoporotic fractures and of colorectal incidence can be achieved [117].

Enrichment of cereal grain products with vitamin D and calcium at indicated levels would be possible within current legal regulations in the USA [117]. It also conforms to legislation introduced in the European Union as of 2007. However, some important member states such as Germany have not yet changed their national law accordingly.

In summary, enrichment with vitamin D and calcium of cereal grain products is an effective and safe measure at very low cost to broaden the range of commonly consumed foods as dietary sources of vitamin D and calcium. This will guarantee at least some modest improvement in both vitamin D and calcium nutrition at the same time without necessitating a change in traditional eating habits. To fulfill individual needs for vitamin D and calcium however, additional consumption of particularly vitamin D- and calcium-rich food and food products or even supplement use is certainly indicated.

## Figures and Tables

**Table 1.** Rating of evidence for association of vitamin D and/or calcium insufficiency with frequent chronic diseases (for details, [1,3]).

**Vitamin D insufficiency**	**Nutritional Calcium deficit**
*A. Convincing evidence from multiple epidemiological (prospective, cross-sectional, retrospective) large cohort studies, interventional trials and experimental studies*	
Osteoporosis	Osteoporosis
Cancer (colorectal, breast)	Cancer (colorectal, breast)
*B. Good evidence from >3 observational studies and/or interventional trials*	
Cancer (renal, prostate, endometrial, ovarian, esophageal, gastric, pancreatic, bladder) Hodgkin’s and non-Hodgkin’s lymphoma	Cancer (renal)
Cardiovascular disease	Cardiovascular disease
	Hypertension
Neuromuscular dysfunctions	Neuromuscular dysfunctions
Diabetes mellitus Type I	
Tuberculosis	
Gingivitis	
Periodontal disease, tooth loss	Periodontal disease, tooth loss
*C. Emerging evidence from observational studies*	
Hypertension	
Metabolic Syndrome	Metabolic Syndrome
Diabetes mellitus Type II	Diabetes mellitus Type II
*D. Evidence mainly from studies with animal models of the respective human disease*	
Inflammatory bowel disease	Inflammatory bowel disease
Multiple Sclerosis	Multiple Sclerosis

**Table 2.** Prevalence of vitamin D insufficiency in healthy adults in selected countries.

**Country**	**Population segment**		**% Vitamin D insufficiency**		**Study**
			**with upper reference limit at**		

	**Age (yr)**	**Gender**	**25–30 nM**	**50 nM**	

***Europe***					
Austria	19–79	M + F	26	60	Kudlacek *et al.* [44]
Denmark	45–58	F	7	40	Brot *et al.* [45]
Finland	31–43	M + F	27		Lamberg–A. *et al.*[47]
France	35–65	M + F	14		Chapuy *et al.* [38]
Germany	18–79	M + F		58	Hintzpeter *et al.* [26]
UK	45	M + F	16	47	Hypponen & Power [46]

***North America***					
Canada (Alberta)	27–89	M + F		61	Rucker *et al.* [52]
USA	20–49	M + F	5	32	Looker *et al.* [51]

***South–East Asia***					
Bangladesh	16–40	F	12 – 17		Islam *et al.* [48]
Japan	30–66	F	10		Nakamura *et al.* [50]

***South Pacific***					
Australia (Queensland)	17–65	M + F	8 a)	23	McGrath *et al.* [49]
Australia (Queensland)	<60	M + F		40	Van der Mei *et al.* [54]
Australia (Victoria)	20–92	F	11	43	Pasco *et al.* [53]
Australia (Geelong)	<60	F		37	Van der Mei *et al.* [54]
Australia (Tasmania)	<60	M + F		67	Van der Mei *et al.* [54]
New Zealand	15–65+	M + F		48	Rockell *et al.* [55]

a) Serum 25-(OH)-D <38 nM

**Table 3.** Prevalence of vitamin D insufficiency in elderly people.

**Country**	**Population segment**		**% Vitamin D insufficiency**		**Study**
			**with upper reference limit of serum 25-(OH)D at**		

	**Age (yr)**	**Gender**	**25–30 nM**	**50 nM**	

***Europe***					
Belgium	76.5 ± 7.5	F	16	43	Neuprez *et al.* [59]
Denmark	70–75	F	17	55	Andersen *et al.* [60]
Finland	70–75	F	10	57	Andersen *et al.* [60]
Ireland	70–75	F	14	60	Andersen *et al.* [60]
Italy	75–80	F	92		v. d. Wielen *et al.* [57]
Netherlands	65+	M + F	13	52	Wicherts *et al.* [58]
Poland	70–75	F	25	92	Andersen *et al.* [60]
Switzerland	75–80	M	12		v. d. Wielen *et al.* [57]

***North America***					
USA	55+	F	4	14	Lappe *et al.* [61]
USA	50–70+	M	10	27	Looker *et al.* [51]

***East Asia***					
Japan	46–82	F	5	28	Nakamura *et al.* [63]

***South Pacific***					
Australia	75+	F	22		Flicker *et al.* [62]
New Zealand	65+	M		41	Rockell *et al.* [55]

**Table 4.** Prevalence of vitamin D insufficiency in children, adolescents and young adults.

**Country**	**Population segment**		**% Vitamin D insufficiency**		**Study**
			**with upper reference limit of serum 25–(OH)D at**		

	**Age (yrs)**	**Gender**	**25–30 nM**	**50 nM**	

***Europe***					
Germany	3–17	M	18	62	Hintzpeter *et al.*[64]
Germany	3–17	F	18	65	Hintzpeter *et al.* [64]
France	13–16	M	78		Guillemant *et al.* [65]
Denmark	12–13	F	51	93	Andersen *et al.* [60]
Finland	12–13	F	37	97	Andersen *et al.* [60]
Ireland	12–13	F	26	89	Andersen *et al.* [60]
Poland	12–13	F	33	87	Andersen *et al.* [60]

***North America***					
USA	12–19	M	10	24	Looker *et al.* [51]
USA	12–19	F	16	31	Looker *et al.* [51]
Canada	18–35	F		>15	Vieth *et al.* [66]

***South–East Asia***					
India (Delhi)	6 – 18	F		91	Puri *et al.* [68]
China	20–35	F	18–40	>90	Woo *et al.* [70]
China	15	F	31	89	Foo *et al.* [69]
Indonesia	18–40	F		63	Green *et al.* [67]
Malaysia	18–40	F		60	Green *et al.* [67]
Japan	19–30	F	42		Nakamura *et al.* [50]

***South Pacific***					
New Zealand	15–18	M		55	Rockell *et al.* [55]
New Zealand	19–24	F		52	Rockell *et al.* [55]

**Table 5.** Nutritional calcium deficit in selected countries.

**Country**	**Age (yrs)**	**DRI a) (mg/day)**	**Calcium intake (mg/day)**		**Study**
			**Gender**		
			**M**	**F**	

***Europe***					
Austria	19–79	>1,000	561 (±290) b)	576 (±309) b)	Kudlacek *et al.* [44]
	<40	1,000	604 (±345) b)	560 (±299) b)	Kudlacek *et al.* [44]
	40–60	>1,000	590 (±318) b)	561 (±287) b)	Kudlacek *et al.* [44]
Germany	18–79	>1,000	1,181 (902–1,535)	1,082 (849–1,379)	Hintzpeter *et al.* [26]
	Adults	1,000	619 (213–1,025)	705 (313–1,094)	Anke [80]
	40–64	>1,000	774 (334–1,330) c)	707 (287–1,225) c)	Schulze *et al.* [81]
UK f)	45–55	1,000	1,133 (950–1,316)	1,063 (931–1,195)	Vyas *et al.* [82]

***North America***					
USA	19–50	1,000	812 (788–837)	626 (596–659)	Ma *et al.* [83]

***South–East Asia***					
Bangladesh	16 – 40	1,000		180 e)	Islam *et al.* [86]
Indonesia	18–40	1,000		270 (239–302) d)	Green *et al.* [67]
Malaysia	18–40	1,000		386 (353–420) d)	Green *et al.* [67]

***South Pacific***					
Australia	20–94	>1,000		643 (±340) b)	Pasco *et al.* [84]

			M + F		

New Zealand	40–64	>1,000	794 (8–1,580) e)	Metcalf *et al.* [85]	

a) Daily Recommended Intake by FAO/WHO [79];

b) mean (±SD);

c) median (90% CI);

d) median (95% CI);

e) mean (range);

f) white, urban population

**Table 6.** Nutritional calcium deficit in elderly people.

**Country**			**Calcium intake (mg/day)**		**Study**

	**Age (yrs)**	**DRI (mg/d)a)**	**M**	**F**	

***Europe***					
Austria	>60	1,300	503 (±221) b)	569 (±287) b)	Kudlacek *et al.* [44]

Belgium	75–80	1,300	748 (324–1,166) c)	676 (287–1,101) c)	Amorim Cruz *et al.* [88]
Denmark	70–75	1,300		544 (127–1,812)d)	Andersen *et al.* [60]
Finland	70–75	1,300		975 (404–2,313) d)	Andersen *et al.* [60]
France	75–80	1,300	620 (402–1,010) c)	635 (428–944) c)	Amorim Cruz *et al.* [88]
Ireland	70–75	1,300		824 (339–1,669) d)	Andersen *et al.* [60]
Netherlands	75–80	1,300	1,036 (725–1,447) c)	1,010(612–1,616) c)	Amorim Cruz *et al.* [88]
Poland	70–75	1,300		325 (86–851) d)	Andersen *et al.* [60]

***North America***					
USA	>55	1,300		611 (381–892) c)	Lappe *et al.* [61]

***South–East Asia***					
Japan	65–75	1,300		527 (±195) b)	Nakamura *et al.* [89]

a) Daily Recommended Intake by FAO/WHO [79];

b) mean (± SD;

c) mean (range);

d) median (97.5% CI)

**Table 7.** Nutritional calcium deficit in children, adolescents and young adults.

**Country**			**Calcium intake (mg/day)**		**Study**

			**Gender**		

	**Age (yr)**	**DRI a) (mg/day)**	**M + F**	**F**	

***Europe***					
Denmark	12.6	1,300		831 (260–2,475) b)	Andersen *et al.* [60]
Finland	12.6	1,300		1,092 (546–2,452) b)	Andersen *et al.* [60]
Ireland	12.6	1,300		728 (54–2,259) b)	Andersen *et al.* [60]
Poland	12.6	1,300		524 (117–1,580) b)	Andersen *et al.* [60]

***North America***					
USA	12.7	1,300	906 (417–1,616) c)		Abrams *et al.* [90]
Canada	18–35	1,000		562 (0–2,630) c)	Rubin *et al.* [91]

***South-East Asia***					
India (Delhi)	6–18	700–1,300		575 (±219) d)	Puri *et al.* [68]

a) Daily Recommended Intake by FAO/WHO [79];

b) median (97.5% CI);

c) mean (range),

d) mean (±SD)
